# Association between PET/CT Scan Findings, Treatment, and Cancer Incidence in a Cohort of AAA Patients

**DOI:** 10.3390/jcm13061569

**Published:** 2024-03-09

**Authors:** Natzi Sakalihasan, Samuel Bruls, Roland Hustinx, Vincent Tchana-Sato, Sarah Sakalihasan, Rebecka Hultgren, Nicos Labropoulos, Alain Colige, Rodolphe Durieux, Pierre Drion, Adelin Albert, Jean-Olivier Defraigne, Lucia Musumeci

**Affiliations:** 1 Department of Cardiovascular and Thoracic Surgery, University Hospital of Liège, 4000 Liège, Belgium; nsaka@chuliege.be (N.S.); s.bruls@chuliege.be (S.B.); vtchanasato@chuliege.be (V.T.-S.); rdurieux@chuliege.be (R.D.); jo.defraigne@chuliege.be (J.-O.D.); 2 Surgical Research Center, GIGA-R Cardiovascular Science Unit, University of Liège, 4000 Liège, Belgium; pvdrion@uliege.be; 3 Division of Nuclear Medicine and Oncological Imaging, Department of Medical Physics, University Hospital of Liège, 4000 Liège, Belgium; rhustinx@chuliege.be; 4 Medical School, University of Liège, 4000 Liège, Belgium; sarah.sakalihasan@student.uliege.be; 5 Karolinska Institutet, Karolinska Universitetssjukhuset, 17176 Stockholm, Sweden; rebecka.hultgren@ki.se; 6 Department of Surgery, Stony Brook University Hospital, Stony Brook, NY 11794-8191, USA; nicos.labropoulos@stonybrookmedicine.edu; 7 Laboratory of Connective Tissues Biology, GIGA-R Cancer Unit, University of Liège, 4000 Liège, Belgium; acolige@uliege.be; 8 Biostatistics and Research Methods Center (B-STAT), University Hospital of Liège, 4000 Liège, Belgium; aalbert@uliege.be

**Keywords:** abdominal aortic aneurysm, FDG PET, survival, cancer, EVAR, endovascular aortic repair

## Abstract

**Background:** Abdominal aortic aneurysm (AAA) is a chronic inflammatory disease that poses several challenges. Given the increasing evidence that AAA patients are more likely to develop cancer and the importance of its early detection, we strived to develop a non-invasive tool based on serial FDG-PET/CT scan examinations to identify, among AAA patients, those at risk of cancer. **Methods:** Between 2006 and 2011 we recruited 149 AAA patients, free of cancer at baseline, and followed them until the end of 2021. All patients underwent an FDG-PET/CT scan at inclusion and possibly more scans during follow-up. At each medical imaging examination, the aneurysmal FDG uptake was recorded. Patients were stratified based on their aortic wall PET status (negative/positive). Any occurrence of cancer was reported. A Cox regression analysis and competing-risk modeling were applied to the data. **Results:** The proportion of AAA patients who developed cancer was 31.5% (mean time to diagnosis was 5.7 ± 3.4 years) and the death rate was 59%. A difference in cancer incidence between PET+ and PET− patients was detected (46.8% vs. 27.3%; HR = 1.96, 95%CI: 1.07–3.57, *p* = 0.028). Moreover, AAA patients undergoing surgical treatment had a lower risk of cancer than unoperated patients (28% vs. 50%; HR = 0.41, 95%CI: 0.21–0.80, *p* = 0.009). **Conclusions:** In AAA patients, diagnostic imaging with an FDG-PET/CT scan can help identify those patients at a higher risk of developing cancer. Moreover, the higher cancer risk in non-surgically treated patients calls for further analysis of associations between aneurysm growth and malignant disease.

## 1. Introduction

Abdominal aortic aneurysm (AAA) is a chronic inflammatory degenerative cardiovascular disease (CVD) promoted by genetic and environmental risk factors such as smoking, aging, Caucasian ethnicity, and male gender. Its prevalence is estimated to range between 1% and 2% in ≥65-year-old men and 0.5% in ≥70-year-old women [1]. Cancer is one of the leading public health concerns worldwide with a high prevalence and death toll. In the European Union, an annual incidence of 4 million new cases of non-melanoma cancers has been recently reported, leading to 1.9 million deaths [2]. Cancer development is in many cases linked to chronic inflammation, dietary factors, obesity, tobacco use, or autoimmunity [3,4]. It is also well known that cancer treatment is associated with an increased risk of CVDs, which has led to the emergence of cardio-oncology as a field of interest for both cardiologists and oncologists [5,6]. More recently, it became evident that, besides the existence of common risk factors, there is a complex and bi-directional interplay between these two types of pathologies, as patients affected by CVDs, including AAA patients, might be at a higher risk of developing cancer [7]. As recent examples, it has been shown that limb arterial thrombosis [8] and heart failure [9,10,11] either promote or are tightly linked to cancer. Further, the concomitant presence of AAA and cancer in retrospective studies was estimated to range between 4% and 38% [12,13,14]. Moreover, we and other authors have long reported a high prevalence of cancer in AAA patients examined with a PET/CT scan with 2-[^18^F]fluoro-2-deoxy-D-glucose (^18^F-FDG) prior to aneurysmal repair [15,16]. Previous studies showed that positive ^18^F-FDG uptake in the aneurysmal wall of AAA patients is associated with inflammatory and phagocytic cell infiltrates [16,17,18,19,20], as well as with a systemic increase in circulating C-reactive protein (CRP) [21] and in metalloproteases proteolytic activity [18,22]. Additionally, such positive uptake has been linked to cellular and molecular alterations prefacing wall deterioration and rupture [21]. On the other hand, it is important to note that arguments against this latter association exist [23] based on concerns about the non-specificity of FDG uptake [24] and on the rarity [25,26] of FDG uptake in AAAs, which are known to be tissues with low cellularity [20]. We further showed that AAA patients with a positive FDG uptake have a specific signature of circulating microRNAs (miRs) [27], which could explain how a localized lesion could induce systemic effects, like cancer development.

Based on these data, a long-term prospective observational study was initiated for AAA patients without previous cancer and also having no cancer at baseline. The main objective of this study was to analyze the risk of developing cancer in AAA patients with either high (PET+ patients) or low (PET− patients) ^18^F-FDG uptake in the aneurysm wall. Moreover, the association between AAA surgical treatment and the later onset of cancer was explored.

## 2. Materials and Methods

### 2.1. Ethical Considerations and Reporting Standard

This study was designed and conducted within the framework of the European program “Fighting Aneurysmal Disease (FAD; B70720095774 and B70720095773 9)” and approved by the ethical committee of the University Hospital of Liège (n°200647 in date 7 February 2011). All participants were informed of the study objectives and signed an informed consent form. This study conformed to the principles outlined in the Declaration of Helsinki and was reported according to the STROBE standards (http://www.strobe-statement.org/strobe-publications, accessed on 1 December 2023).

### 2.2. Cohort

The study population, recruited between 2006 and 2011 at the Department of Cardiovascular Surgery, University Hospital Center (CHU) of Liège, Liège, Belgium, included 149 AAA patients followed by assessing the occurrence of cancer, death, or the end of follow-up (31 December 2021), whichever came first. An AAA diagnosis was confirmed with an ultrasound exam. Patients entering the study underwent a first ^18^F-FDG-PET/CT scan examination, which was eventually repeated later in the context of their AAA management. The exclusion criteria were having cancer at the time of inclusion (prevalence cases) or having a history of cancer. In a recently submitted manuscript, Bruls et al. report that female patients have a higher incidence of positive PET/CT scans compared to males (submitted to JCM). In this cohort, the number of available female patients was small (10% of the total cohort). Considering these factors, women were not included in our study cohort since it would not be possible to do an adequate sub-group stratified analysis based on sex differences.

### 2.3. Baseline Characteristics and Risk Factors

Within the framework of a European program, at inclusion, each eligible AAA patient performed at least one ^18^F-FDG-PET/CT scan, and the following characteristics and AAA risk factors were recorded: age, smoking (past + current smokers vs. never smokers), and a history of diabetes, hypertension, chronic obstructive pulmonary disease (COPD), renal insufficiency (RI), stroke, hyperlipidemia (HLD), acute myocardial infarction (AMI), peripheral artery disease (PAD), and angina pectoris.

### 2.4. Definitions

Patient follow-up (FU) included a PET/CT scan regardless of the abdominal aortic diameter. Moreover, independently from the PET/CT results, but according to the AAA diameter, patients were either non-operated (NOP) or underwent open surgical repair (OSR) or endovascular aortic repair (EVAR). Cancer development and treatment (surgical, chemotherapy, radiotherapy, surgical + chemotherapy, surgical + radiotherapy, surgical + immunotherapy, informed refusal, or unknown), together with their FDG-PET status, were recorded. Some cancers were diagnosed by a PET/CT performed during the AAA FU. Each cancer was confirmed by a histological analysis from a biopsy or a specimen that was obtained during surgery. All reported cancers were incidence cases.

For PET/CT scan image acquisition, patients were asked to fast for at least 6h before injecting ^18^F-FDG through an indwelling catheter. After an uptake time of 60 min on average, the PET/CT scan acquisition was started, according to a protocol fully described in Courtois A, et al. [21,28]. All PET/CT scans were independently evaluated by specialized doctors. The radioactive signal accumulating in the tissue was reported as the Standard Uptake Value (SUV), a parameter correction for the activity of the product injected and the body mass of the patient. The SUV_max_ in the region of interest and the abdominal aorta were then normalized to the liver SUV to give the relative SUV (SUVr) or SUV ratio. A patient’s PET status was classified as “positive” (PET+) when at least one ^18^F-FDG PET/CT scan had an SUVr ≥ 1 from the inclusion in the study until the occurrence of death or the last follow-up assessment; otherwise, the patient’s PET status was classified “negative” (PET−). All the PET/CT scan analyses were performed in the context of the AAA follow-up. These preliminary imaging studies also confirmed the absence of significant malignant lesions.

### 2.5. Outcomes

The primary outcome measure was the time-to-occurrence of cancer. Secondary outcomes included the types of cancer, presence of metastasis, and survival time. In the case of death, the cause was recorded as follows: AAA, cancer, pulmonary, cardiac, cerebral, other, or unknown. The time-to-cancer and time-to-death were censored on 31 December 2021.

### 2.6. Statistical Analysis

Quantitative data were expressed as the mean and standard deviation (SD) or as the median and interquartile range (IQR). Frequency tables (numbers and percentages) were used for binary and categorical factors. The cumulative incidence rate of cancer was estimated by the competing risk model, while the overall survival (OS) was estimated by the Kaplan–Meier (KM) method. To account for time-varying PET SUVr values, a Cox proportional hazard regression model with time-dependent covariates was applied to determine the effect of risk factors on cancer occurrence (cancer-free survival) and OS [29]. Specifically, 284 time-intervals were defined up to cancer occurrence and 290 intervals up to death. Only the PET before the event was taken into account for the model. The association between time-to-event variables and covariates was assessed by the hazard ratio (HR) and its 95% confidence interval (95%CI). Results were considered significant at the 5% critical level (*p* < 0.05). All statistical calculations were performed with SAS (version 9.4) and R (version 3.5) software packages.

## 3. Results

### 3.1. Patient Characteristics

**Figure 1:**
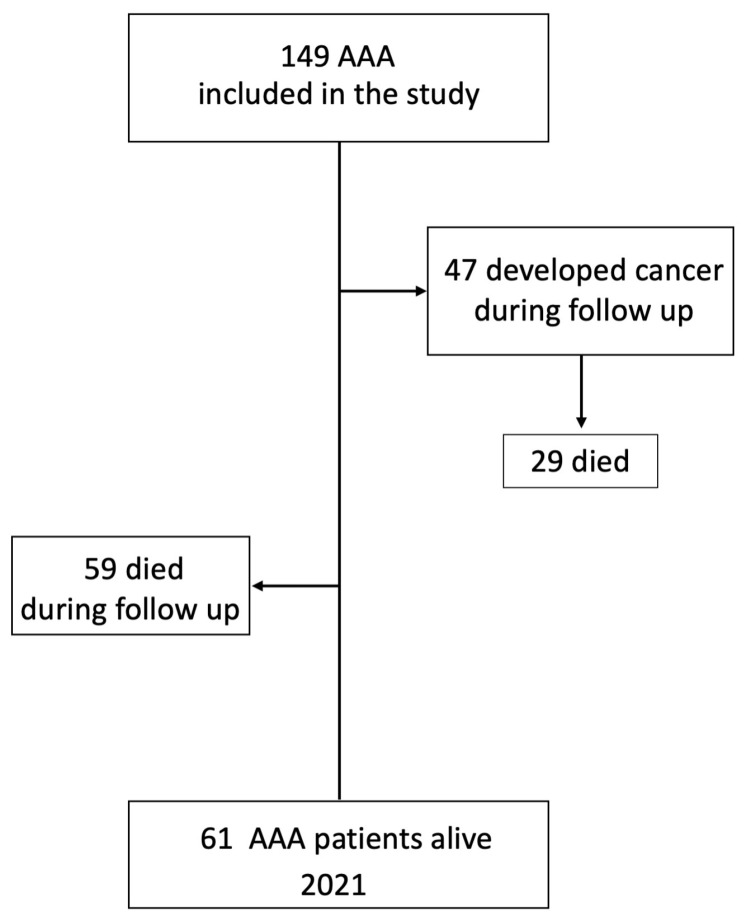
Flowchart of patient selection.

The cohort consisted of 149 AAA male patients all free of any malignancy, as evidenced by PET/CT imaging performed upon admission to the study (Figure 1).

The mean age at inclusion was 71.5 ± 8.8 years and the mean age at AAA diagnosis was 68.9 ± 9.1 years, yielding a mean delay of 2.6 ± 3.4 years. Smoking was the most prevalent risk factor (85.9%), followed by hyperlipidemia (65.1%) and hypertension (61.1%) (Table 1).

### 3.2. Cancer Incidence during Follow-Up

The median FU time was 8.5 years (IQR: 4.7–11.5) with a maximum of 15.5 years. A total of 47 patients developed cancer, yielding an incidence rate of 31.5%. The cumulative incidence rate of cancer, estimated by the competing risk model, is displayed in Figure S1. Among these cancer cases, 17 (36.2%) developed metastases (Table 2). Cancer occurred on an average of 5.7 ± 3.4 years from inclusion, and the mean age of the patient diagnosed with cancer was 77.6 ± 8.6 years. The most common cancers were urologic (29.8%), followed by lung cancer (27.7%). Most cancer patients were treated surgically (53.2%, including combination treatment).

### 3.3. PET Positive Status of a Patient Is Associated with Cancer Development

**Figure 2:**
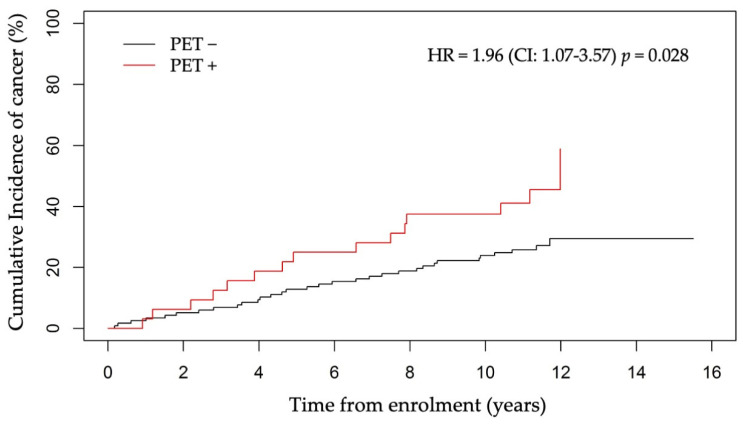
Cumulative incidence rate of cancer in PET+ and PET− patients with competing risk adjustment.

All patients had a PET/CT scan at inclusion, with 85 patients (57%) undergoing at least one additional PET/CT scan during FU (Table S1). In total, 291 PET/CT scans were performed during the study period, yielding about 2 scans/patient. The mean delay between the first and last PET/CT scan was 1.9 ± 1.0 years. The PET status of a patient was defined as “positive” (PET+) when at least one PET/CT scan had SUVr ≥ 1 and “negative” (PET−) otherwise (see Material and Methods). Globally, there were 32 (21.5%) PET+ patients and 117 (78.5%) PET− patients. Among the PET+ patients, 15 developed cancer (46.8%), while this percentage was markedly reduced in PET− patients (27.3%, 32 patients) (Table S2). The cumulative incidence rate of cancer, determined using the competing risk modeling approach, differed significantly between PET+ and PET− patients (HR = 1.96, 95%CI: 1.07–3.57, *p* = 0.028), as displayed in Figure 2.

### 3.4. Risk Factors Associated with Cancer Incidence

No association was found between smoking and cancer (83.0%, 39 smokers among 47 patients that developed cancer and 87.2%, 89 smokers among 102 patients that did not develop cancer, *p* = 0.61)**,** as well as between smoking and PET status (*p* = 0.40) (Supplemental Table S2). These findings were confirmed by a univariate or multivariate Cox regression analysis with a time-varying PET (Table 3).

By contrast, PET positivity and old age were both associated with an increased risk of developing cancer. Thus, being older and/or having a positive PET increases the risk of detecting cancer.

### 3.5. AAA Surgery and Cancer Incidence

**Figure 3:**
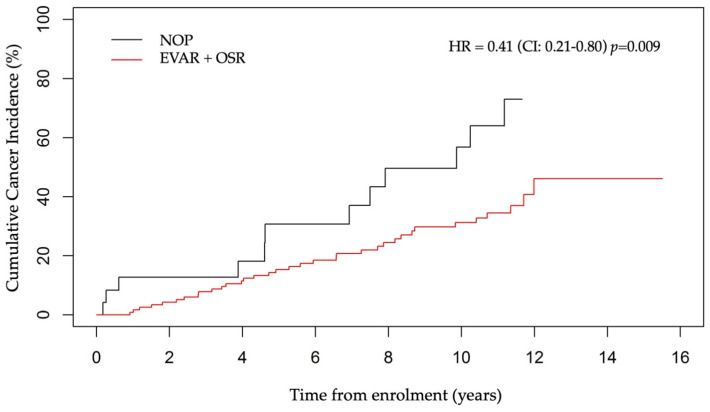
Cumulative cancer incidence in EVAR + OSR vs. NOP group of patients.

AAA patients in the cohort were either treated by EVAR or OSR, or neither for NOP patients. The majority of the patients (125/149, 84%) underwent surgical treatment (EVAR + OSR). The mean age of patients at surgical intervention was 73.6 ± 8.8 years, which was on average 4.6 ± 4.2 years after their AAA diagnosis. The incidence of cancer in NOP patients was higher than in operated patients. Specifically, 50% of NOP subjects developed cancer (12 on 24 NOP), against 34% who underwent an EVAR (12 on 35 EVAR), 26% who underwent an OSR (23 on 90 OSR) and 28% who underwent an EVAR + OSR (35 on 125 EVAR + OSR) (HR = 0.41, 95%CI: 0.21–0.81, *p* = 0.009) (Figure 3).

While the difference remained significant between the OSR and NOP groups (HR = 0.39, 95%CI: 0.19–0.79, *p* = 0.008) (Figure S2a), only a tendency was noted between the EVAR and NOP groups (HR = 0.48, 95%CI: 0.21–1.09, *p* = 0.081) (Figure S2b), and no difference between the OSR and EVAR groups (graph not shown). The cumulative cancer incidence, when considering the three groups, is shown in Figure S2c.

### 3.6. Cancer Incidence According to PET Status and AAA Treatment

Although the proportions of PET− vs. PET+ patients were comparable in each treatment group (*p* = 0.75) (Table S3), the cancer incidence in each treatment group changed according to PET status; the difference was only significant in the group of non-operated patients (Table S4). Specifically, among the 24 NOP patients, all 5 PET+ patients (100%) developed cancer (*p* = 0.04), while in the EVAR group, the cancer incidence was 50% among the PET+ patients (*p* = 0.39) and 33% among the PET+ subjects belonging to the OSR group (*p* = 0.40).

### 3.7. Overall Survival

The death rate in the cohort was 59% and the deaths occurred at the mean age of 79.7 ± 9.0 years. The cancer incidence was unrelated to mortality (*p* = 0.72) (Table S5 and KM curves in Figure S3).

Among the 47 cancer patients, 29 died (62%) mainly from cancer itself (72%), or from a cardiovascular condition (7%), AAA (3%), or other or unknown causes (17%). Among the 102 patients without cancer, 59 died (58%), due to a cardiovascular condition (27%), then pulmonary (9%), cerebral (9%), AAA (7%), and 49% due to other or unknown causes. Globally, the most common cause of death was cancer (24%), then cardiac-related diseases (21%), cerebral (6%), pulmonary problems (6%), and AAA (5%) (Table S6).

A higher death rate was seen among PET+ patients (68.8%, 22 patients out of 32) than PET− patients (56.4%, 66 patients out of 117), but the difference was not significant (see also KM survival curves in Figure S4).

### 3.8. Risk Factors Associated with Survival

Univariate and multivariate Cox regression analyses with a time-varying PET were used to assess the risk factors potentially associated with survival (Table 4).

The time-varying PET turned out to be only significant in the multivariate model, jointly with age and COPD. Some risk factors like RI, AMI, and angina pectoris were significant in the univariate model but were not when combined in a multivariate setting. Of note, the time from AAA diagnosis to entry increased the risk of mortality only in the multivariate analysis with a time-varying PET.

## 4. Discussion

This long-term prospective observational study was set up to evaluate the general prognostic value of PET positivity of the aneurysmal wall in AAA patients. The cancer risk was found to be higher in the PET+ patients (~50% of PET+ patients developed cancer against ~30% in the PET− group). Areas in the aortic wall with a high FDG uptake are associated with inflammation, aortic wall instability, acute symptoms [30], as well as with complications after EVAR [28]. Our hypothesis was that having just one PET scan positive during FU would be sufficient to potentially cause long-term effects, like generating favorable conditions for cancer development. On the other hand, we have also used a time-varying PET analysis, which takes into account only scans before cancer occurrence, to determine the association between PET, cancer, and risk factors. Among risk factors, only age was strongly and independently associated with cancer occurrence, while smoking was not associated with cancer or with PET status.

The incidence rate of cancer in our cohort was 31.5%, which is markedly higher compared to the general Belgian population, or to a cohort of coronary artery disease (CAD) patients (17.8%) in our teaching hospital (unpublished data, presented by N. Sakalihasan during the 6th International Meeting on Aortic Disease (IMAD) on 12–14 September 2018 and manuscript in preparation).

These observations might suggest that AAA is a carcinogenic condition, or accelerates the growth of primordial lesions, probably due to the inflammatory nature of AAA.

Indeed, the two diseases are considered chronic inflammatory diseases, with many cancers triggered by chronic inflammation secondary to dietary factors, obesity, tobacco use, autoimmunity, or chronic infection [2,31].

As an additional link between AAA, inflammation, and cancer, we have found that the suppression of the local inflammation due to AAA by surgical treatment markedly reduced cancer occurrence, information that might change the clinical management of AAA patients in the future. Other data point to a causal link between AAA and cancer. In NOP patients, PET+ status was a better predictor of cancer occurrence than in EVAR and OSR subjects, probably due to the suppression of the inflammatory zone by surgery. Further, the fact that AAA patients undergoing OSR developed less cancer (26%) than non-operated patients (50%) does support the idea that local AAA inflammation can lead to cancer in people with a certain predisposition. Similarly, the slightly higher risk of cancer in EVAR patients than in OSR patients could be related to the fact that the aneurysm sac is still present after an EVAR, which could lead to the persistence of a low-grade inflammatory process. Therefore, local inflammation (detected by a PET+ signal) associated with a systemic biomarker signature should alert to the possible presence or future development of cancer.

While previous studies linking AAA and cancer were mainly associative, our study provides a proof-of-concept of a potential causal link between AAA and cancer, but it does not determine whether AAA is likely to induce cancer de novo rather than accelerate tumor growth from pre-existing, pre-cancerous cells, or from very small, low-proliferative tumors. Finally, improved knowledge of signaling pathways of cancer occurrence in AAA patients could initiate the development of specific drugs targeting the newly discovered pathways. To clarify the common pathways dysregulated in both diseases, and determine their pathogenesis and potential causal link between AAA and cancer, an in-depth analysis of local and systemic biomarkers common to AAA and cancer will be necessary. Interestingly, some of the factors involved in AAA formation and progression, such as growth factors, proteinases, matrikines, chemokines, and ROS [1,21,32,33], are also recognized players in cancer development and progression [34,35].

It has been reported that an EVAR or OSR in AAA patients with concomitant cancer, has a poor outcome and that AAA patients with a history of cancer undergoing repair have a mortality rate higher than those without any cancer history (21.21% vs. 17.08%, HR = 1.31, 95% CI: 1.17–1.46, *p* < 0.0001) [36]. In our cohort of cancer-free AAA patients, surgical treatment (EVAR or OSR) improved survival compared to NOP patients, but not significantly (58% mortality in EVAR + OSR vs. 67% in NOP *p* = 0.67).

Cancer patients were not at a higher risk of death, as shown by KM curves; although, in the whole AAA cohort, the most common cause of death was cancer (24%). Although the KM curves show that PET status was unrelated to survival, on the other hand, a time-varying PET turned out to be independently associated with survival in a multivariate analysis, together with age and COPD. This can be explained by the fact that PET status takes into account all the PET/CT scans performed while a time-varying PET analysis considers the PET/CT scan just before death.

The finding that the most common cause of death in the AAA cohort is cancer challenges the focus on the risk of cardiovascular events in AAA patients.

## 5. Study Limitations

Our study is constrained by a relatively small sample size, precluding the inclusion of female AAA patients. Considering this limitation, we encourage multicenter studies which will allow a better understanding of sex-specific differences.

## 6. Conclusions

Two main findings result from our study. First, we have shown that PET positivity of AAA, reflecting local aortic inflammation, is associated with an increased cancer risk. Our data also indicate that the surgical treatment of AAA, which reduces inflammation, is associated with lower cancer occurrence. These findings will need to be supported by larger studies and the inclusion of translational explorations, including analyses of aneurysmal walls and cancers forming in AAA patients. Despite this limitation, we are convinced that our data could guide physicians in the long-term treatment of AAA patients, and make them aware that cancer and AAA, two diseases involving inflammatory processes, have probably common pathogenic mechanisms.

## Figures and Tables

**Table 1:** Baseline characteristics of AAA patients (*n* = 149).

Variable	Mean ± SD Number (%)
Age (years)	71.5 ± 8.8
Smokers	128 (85.9)
Diabetes	20 (13.4)
Hypertension	91 (61.1)
COPD	52 (34.9)
RI	22 (14.8)
Stroke	20 (13.4)
HLD	97 (65.1)
AMI	46 (30.9)
PAD	41 (27.5)
Angina pectoris	22 (14.8)

Smokers include past and current smokers; COPD: chronic obstructive pulmonary disease; RI: renal insufficiency; HLD: hyperlipidemia; AMI: acute myocardial infarction; PAD: peripheral artery disease.

**Table 2:** Cancer incidence, type, and treatment among AAA patients (*n* = 149).

Variable	Number of Patients	Number of Cases (%)
Cancer Incidence	149	47 (31.5)
Metastasis	47	17 (36.2)
Type of Cancer	47	
Urologic		14 (29.8)
Lung		13 (27.7)
Digestive		9 (19.1)
Skin		5 (10.6)
Acute myeloid leukemia		2 (4.3)
Other		4 (8.5)
Cancer treatment	47	
Surgical		20 (42.6)
Chemotherapy		5 (10.6)
Radiotherapy		4 (8.5)
Surgical + Radiotherapy		3 (6.4)
Surgical + Immunotherapy		1 (2.1)
Surgical + Chemotherapy		1 (2.1)
Informed Refusal		10 (21.3)
Unknown		3 (6.4)

**Table 3:** Univariate and multivariate Cox regression analysis of risk factors for cancer occurrence with time-varying PET/CT scans.

	Univariate Model			Multivariate Model		
Covariate	HR	HR (95%CI)	*p*-Value	HR	HR (95%CI)	*p*-Value
Age at baseline (years)	1.03	1.00–1.07	0.047	1.04	1.00–1.08	0.042
AAA diagnosis since entry (years)	0.99	0.91–1.08	0.86	0.99	0.91–1.09	0.89
Smoking (Yes vs. No)	0.86	0.40–1.86	0.70	0.90	0.40–2.03	0.80
Diabetes (Yes vs. No)	1.02	0.45–2.32	0.96	0.89	0.37–2.13	0.78
Hypertension (Yes vs. No)	0.88	0.49–1.57	0.66	0.92	0.50–1.68	0.78
COPD (Yes vs. No)	1.06	0.56–2.02	0.86	1.03	0.51–2.05	0.94
RI (Yes vs. No)	0.72	0.26–2.01	0.53	0.64	0.22–1.87	0.41
Stroke (Yes vs. No)	0.61	0.22–1.71	0.35	0.55	0.19–1.61	0.28
HLD (Yes vs. No)	0.81	0.44–1.48	0.49	0.86	0.46–1.61	0.49
AMI (Yes vs. No)	0.82	0.42–1.63	0.57	0.72	0.33–1.57	0.40
PAD (Yes vs. No)	1.04	0.54–2.01	0.91	1.19	0.57–2.51	0.64
Angina Pectoris (Yes vs. No)	1.14	0.48–2.71	0.76	1.24	0.46–3.36	0.67
Time-varying PET (+ vs. −)	2.34	1.18–4.64	0.015	2.34	1.12–4.92	0.025

**Table 4:** Univariate and multivariate Cox regression analysis of risk factors for survival with time-varying PET.

	Univariate Model			Multivariate Model		
Covariate	HR	HR (95%CI)	*p*-Value	HR	HR (95%CI)	*p*-Value
Age at baseline (years)	1.07	1.04–1.10	<0.0001	1.07	1.04–1.11	<0.0001
AAA diagnosis to entry (years)	0.96	0.89–1.03	0.21	0.93	0.87–1.00	0.062
Smoking (Yes vs. No)	0.96	0.53–1.74	0.90	0.81	0.42–1.56	0.53
Diabetes (Yes vs. No)	0.74	0.38–1.45	0.38	0.71	0.35–1.44	0.34
Hypertension (Yes vs. No)	1.07	0.69–1.64	0.77	1.12	0.71–1.78	0.63
COPD (Yes vs. No)	2.02	1.32–3.09	0.0011	2.12	1.34–3.35	0.0014
RI (Yes vs. No)	1.77	1.02–3.06	0.042	1.36	0.74–2.50	0.32
Stroke (Yes vs. No)	1.40	0.79–2.50	0.25	1.39	0.75–2.56	0.29
HLD (Yes vs. No)	0.71	0.46–1.10	0.13	0.80	0.51–1.27	0.34
AMI (Yes vs. No)	1.63	1.05–2.52	0.029	1.52	0.93–2.47	0.097
PAD (Yes vs. No)	1.20	0.76–1.90	0.43	1.44	0.87–2.40	0.16
Angina Pectoris (Yes vs. No)	1.98	1.17–3.33	0.010	1.37	0.76–2.45	0.29
Time-varying PET (+ vs. −)	1.56	0.92–2.66	0.099	2.33	1.30–4.20	0.0048

## Data Availability

The data presented in this study are available on request from the corresponding author.

## Supplementary Materials

The following supporting information can be downloaded at: https://www.mdpi.com/article/10.3390/jcm13061569/s1, Figure S1: Cumulative incidence rate for cancer, estimated by competing risk model; Figure S2: Cumulative cancer incidence in NOP, OSR, and EVAR group of patients; Figure S3: Overall survival in Cancer− vs. Cancer + patients (KM curves); Figure S4: Overall survival in PET− vs. PET+ patients (KM curves); Table S1: Distribution of the number of PET/CT scans performed per patient; Table S2: Distribution of patients according to PET status (positive/negative) and cancer occurrence or PET status and smoking; Table S3: Distribution of patients according to AAA treatment and PET status; Table S4: Distribution of patients according to AAA treatment, cancer occurrence and PET status; Table S5: Distribution of patients according to outcome and cancer occurrence; Table S6: Distribution of causes of death.
