# Supplementary material for: Association between PET/CT Scan Findings, Treatment, and Cancer Incidence in a Cohort of AAA Patients
Source: J Clin Med. 2024 Mar 9;13(6):1569. doi: 10.3390/jcm13061569 (PMC10970866; doi:10.3390/jcm13061569)
Supplement: Supplementary file 1 [file jcm-13-01569-s001.zip › jcm-2892929-supplementary.pdf]

Journal of
Clinical Medicine

Supplemental Material

Supplemental Figures

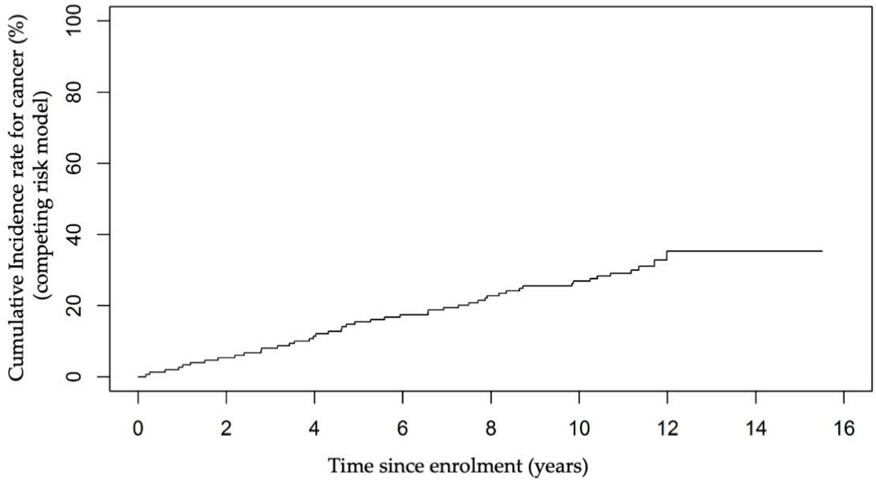

Supplemental Figure S1. Cumulative incidence rate for cancer, estimated by competing risk model.

a.

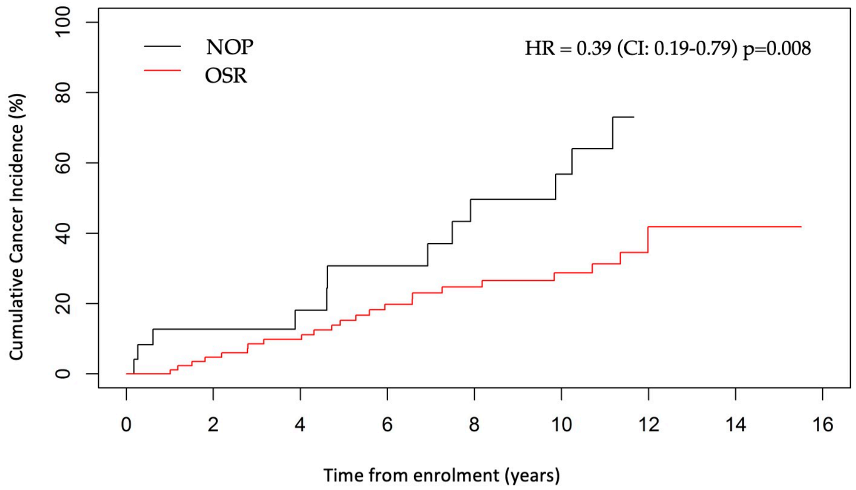

b.

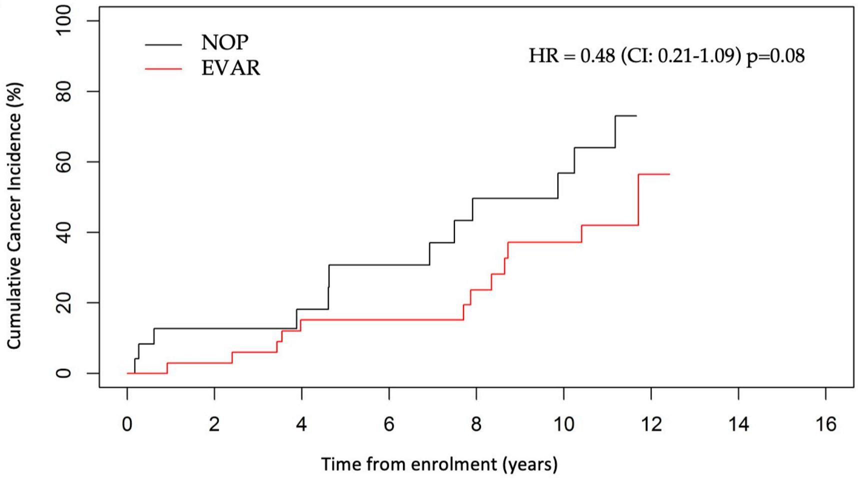

c.

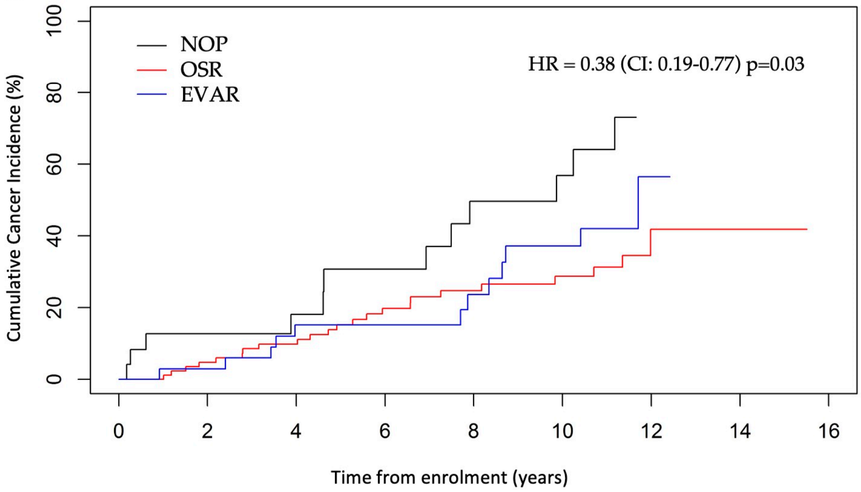

Supplemental Figure S2. Cumulative cancer incidence in NOP, OSR and EVAR group of patients. Comparison of two groups, OSR vs NOP **(a)** or EVAR vs NOP **(b)** or three groups OSR vs EVAR vs NOP **(c)**.

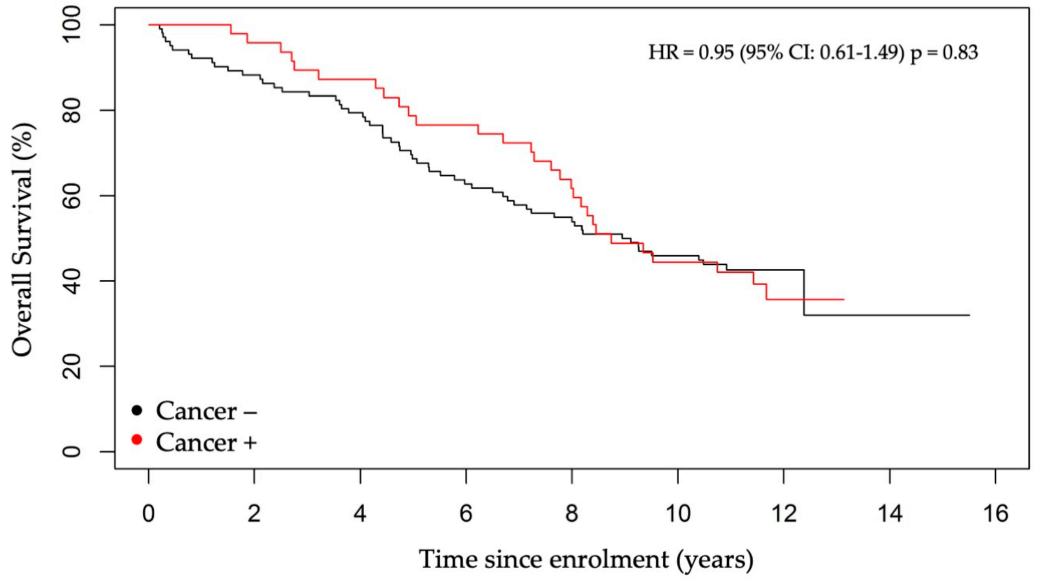

Supplemental Figure S3. Overall survival in Cancer – versus Cancer + patients (KM curves).

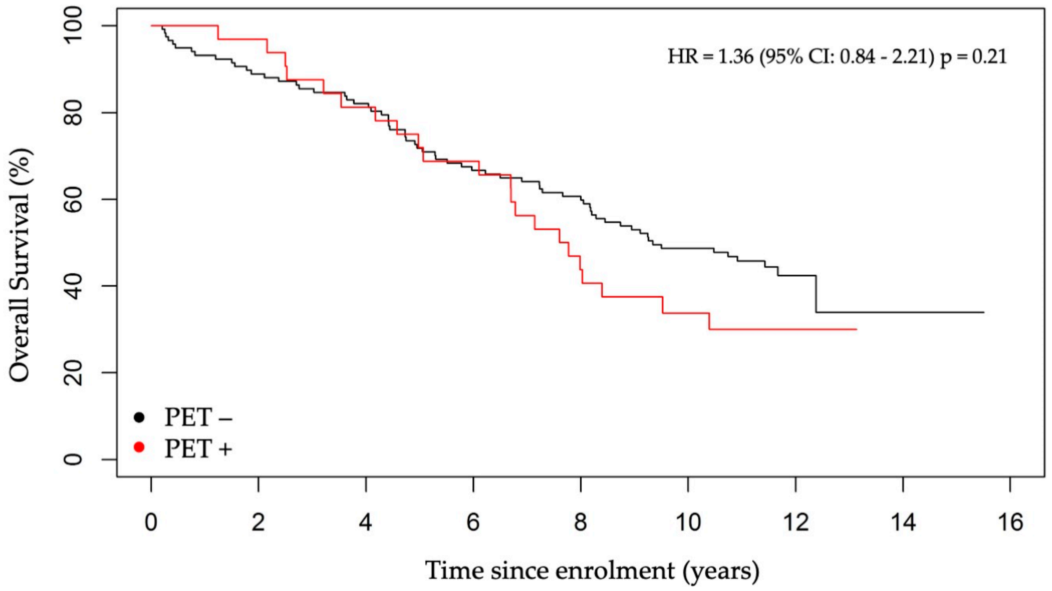

Supplemental Figure S4. Overall survival in PET – versus PET + patients (KM curves).

Supplemental Tables

Supplemental Table S1. Distribution of the number of PET/CT scans performed per patient.

Number of PET/CT per patient	Number of patients (%)	Number of scans (%)	Number of patients with SUVr≥1 (%)	Number of patients PET + and Cancer +
1	64 (43.0)	64 (22.0)	8 (12.5)	5 (62.5)
2	43 (28.9)	86 (29.6)	7 (16.3)	4 (57.1)
3	29 (19.5)	87 (29.9)	13 (44.8)	4 (30.8)
4	11 (7.4)	44 (15.1)	3 (27.3)	1 (33.3)
5	2 (1.3)	10 (3.4)	1 (50.0)	1 (100.0)
Total	149	291 (21.5)	32 (21.5)	15 (46.9)

Supplemental Table S2. Distribution of patients according to PET status (positive/negative) and cancer occurrence or PET status and smoking. P-value was calculated by Fisher exact test.

**Variable**	**PET +**	**PET -**	***p*-value**
	**n (%)**	**n (%)**	
Cancer	15 (46.8)	32 (27.3)	0.03
Smoking	26 (81.3)	102 (87.2)	0.40
Total (*n*=149)	32 (21.5)	117 (78.5)	

Supplemental Table S3. Distribution of patients according to AAA treatment and PET status

Variable	NOP n (%)	EVAR n (%)	OSR n (%)	*p*-value
PET +	5 (20.8)	6 (17.1)	21 (23.3)	0.75
Total (n=149)	24 (16.1)	35 (23.5)	90 (60.4)	

Supplemental Table S4. Distribution of patients according to AAA treatment, cancer occurrence and PET status

Variable	PET + n (%)	PET - n (%)	*p*-value
Cancer in NOP	5/5 (100)	7/19 (37)	0.04
Cancer in EVAR	3/6 (50)	9/29 (31)	0.39
Cancer in OSR	7/21 (33)	16/69 23)	0.40

Supplemental Table S5. Distribution of patients according to outcome and cancer occurrence

Variable	Dead n (%)	Alive n (%)	*p*-value
Cancer	29 (33.0)	18 (29.5)	0.72
Total (n=149)	88 (59.1)	61 (40.9)	

Supplemental Table S6. Distribution of causes of death

Causes of death	Number of Patients (% total n=149; % dead n=88)
Cancer	21 (14.1; 23.9)
AAA	5 (3.4; 5.7)
Cardiac	18 (12.1; 20.5)
Pulmonary	5 (3.4; 5.7)
Cerebral	5 (3.4; 5.7)
Other*	22 (14.8; 25.0)
Unknown	12 (8.1; 13.6)
Total	88 (59.1)

*other causes include: mainly old age, then sepsis, orthopedic surgery complications, renal insufficiency, intestinal occlusion.
